# A Bayesian mixture model approach to examining neighbourhood social determinants of health in endometrial cancer care in Massachusetts

**DOI:** 10.1093/jrsssa/qnag047

**Published:** 2026-03-30

**Authors:** Carmen B. Rodríguez, Stephanie M. Wu, Stephanie Alimena, Alecia J. McGregor, Briana J. K. Stephenson

**Affiliations:** 1 Department of Biostatistics, Harvard T.H. Chan School of Public Health, Boston, MA, USA; 2 Public Health Data Science, Division of Psychiatry, University College London, London, UK; 3 Division of Gynecologic Oncology, Department of Obstetrics and Gynecology and Reproductive Biology, Brigham and Women’s Hospital, Boston, MA, USA; 4 Department of Obstetrics, Gynecology, and Reproductive Biology, Harvard Medical School, Boston, MA, USA; 5 Department of Health Policy and Management, Harvard T.H. Chan School of Public Health, Boston, MA, USA

**Keywords:** Bayesian clustering, disparities, endometrial cancer, national comprehensive cancer network, neighbourhoods, social determinants of health

## Abstract

Many studies examine social determinants of health (SDoH) in isolation, overlooking their interconnected nature. We used a multifactorial approach to construct a neighbourhood-level measure that explores how SDoH jointly impact care received for endometrial cancer (EC) patients in Massachusetts (MA). Using 2015–2019 American Community Survey data, we applied a Bayesian multivariate Bernoulli mixture model to identify MA neighbourhoods with similar SDoH characteristics. Five neighbourhood SDoH (NSDoH) profiles were derived and characterized: (1) advantaged non-Hispanic White; (2) disadvantaged racially/ethnically diverse, more renter-occupied housing with limited English proficiency; (3) working class, lower educational attainment; (4) racially/ethnically diverse and greater economic security and educational attainment; and (5) racially/ethnically diverse, more renter-occupied housing with limited English proficiency. We assigned these profiles to EC patients in the Massachusetts Cancer Registry and used them as the main exposure in a Bayesian logistic regression, adjusting for sociodemographic and clinical characteristics. NSDoH profiles were not associated with optimal care; however, patients in all other profiles had lower odds compared to Profile 1. Our findings demonstrate how a flexible model-based clustering approach captures the multidimensional nature of NSDoH in an interpretable way and may support targeted public health interventions based on neighbourhood-specific social factors to improve healthcare delivery.

## 1 Background

Social determinants of health (SDoH) are defined as the social or demographic components of one’s environment that shape health outcomes and can impact an individual’s access to care, treatment decisions, and overall patient experiences (Cooper et al., 2024; Coughlin, 2019; Kolak et al., 2020). These determinants, including socioeconomic status, experiences of racism or discrimination, and neighbourhood conditions, can introduce bias in the healthcare system, potentially leading to a lack of guideline-concordant treatment (Gomez et al., 2015), such as for endometrial cancer (EC), one of the most commonly occurring female cancers. Despite the existence of evidence-based treatment guidelines, disparities across the EC care continuum persist, particularly among ethnically and socioeconomically disadvantaged groups (Doll, 2023; Doll et al., 2018; Eakin et al., 2023; Huang et al., 2020; Karia et al., 2023; Makker et al., 2021; Paskett & Bernardo, 2020; Rodriguez et al., 2021a, 2021b). This disparate relationship serves as our motivation to better understand how SDoH patterns are considered and characterized (Eakin et al., 2023; Makker et al., 2021). A comprehensive analysis of SDoH that transcends race and ethnicity would provide researchers and policymakers with a deeper understanding of how the social environment can influence a patient’s ability to engage with the healthcare system (Rodriguez et al., 2021a, 2021b).

Characterization of SDoH is often conducted using nonstandardized composite scores of proxy variables that correspond to different domains, such as income, education, and employment (Jonnalagadda et al., 2020; Rethorn et al., 2020). At the geographic level, SDoH has been operationalized using area-based measures derived from publicly available data sources such as the United States (US) Census and the American Community Survey (ACS). Two commonly used measures are the Yost Index and the Area Deprivation Index (ADI) (Berg et al., 2021; Singh, 2003; Yost et al., 2001). The Yost Index, typically used in national cancer surveillance, operates under the assumption that SDoH is one-dimensional. It inputs seven area-level variables (median household income, education, proportion of households below the 150% poverty line, median house value, median gross rent, unemployment rate, and working-class occupation). Summarized at the census tract or block level, these variables are clustered via a principal component analysis (PCA), based on how much the respective variable explains the variation in the data (Boscoe et al., 2021; LaFantasie & Boscoe, 2022; Yost et al., 2001). Referred to as the primary component, the socioeconomic status (SES) index is calculated for each areal unit based on the variable loadings on that component. Areal units are then ranked based on their SES scores and divided into quintiles, with quintile 1 representing the lowest SES and quintile 5 the highest. As a one-dimensional component for SES, a loss of information results because this measure focuses on the one or two variables that may be most relevant to classifying low or high SES, as opposed to considering the combination of the different variables and how their contributions may differ from one residential area to another.

The ADI, originally developed in 2002 to study general mortality disparities and hospitalizations in the US, expands the set of census-derived SDoH variables to 17. Previously, it operated under the same one-dimensional assumption as Yost, but now incorporates a three-factor model to accommodate multidimensionality (Berg et al., 2021; Boscoe et al., 2021; Kind & Buckingham, 2018; Singh, 2003). Highly correlated variables are first clustered together and then defined along the following three domains: financial strength, economic hardship and inequality, and educational attainment (Berg et al., 2021). As in the Yost Index, areal units are indexed based on how high they load on the three-factor domains. Higher percentile ranks indicate greater socioeconomic disadvantage. A limitation of the ADI is that the derived domains are treated independently from one another without acknowledging the ways in which they intersect. Boscoe et al. (2021) compared the Yost and ADI indices and found that, despite being derived from the same data and heavily influenced by financial domains, they differ significantly in terms of transparency for reproducibility, distribution, and sensitivity to missing data. Methodologically, these approaches share similarities in their use of principal components or factor analysis to reduce the number of highly correlated SDoH variables, but the domains derived are treated independently from one another and fall short of acknowledging the ways in which they intersect.

Recently, Kolak et al. (2020) introduced a new approach that further expands the list of census-derived SDoH variables for PCA, including variables like limited English proficiency. These variables were reduced to four domains: socioeconomic advantage index, limited mobility index, urban core opportunity index, and mixed immigrant cohesion and accessibility index. Additionally, they clustered census tracts by characterizing them into profiles based on similar scoring across the four SDoH components using K-means clustering. While this approach does allow for clustering based on shared responses to the different domains, the clustering is based on a single summary score. Overlooking how each component-specific score contributes to the summary score ignores the heterogeneity present in area deprivation. Two neighbourhoods could potentially be clustered together but experience different patterns of resource allocation, which alter the level of impact these residents may experience in regards to their access and engagement with the healthcare system. New methodological approaches are imperative to accommodate the interconnectedness and multifactorial structure of SDoH, inclusive of factors pertinent to vulnerable populations that are at greater risk of health disparities (Boscoe et al., 2021; Kolak et al., 2020; Rethorn et al., 2020; Zhang et al., 2024).

Model-based clustering, specifically finite mixture models (FMM), are able to identify underlying patterns in heterogeneous populations from a wide set of interrelated variables. Lekkas et al. recently highlighted the methodological advantages of FMMs in neighbourhood health research through the common application of latent class analysis (LCA) and latent profile analysis (LPA). These models are able to characterize neighbourhood profiles based on a set of multivariate categorical or continuous data and examine their association with various health indicators (Lekkas et al., 2019). Although LCA or LPA are well-suited to handle the multifaceted nature of neighbourhoods, typically, the number of latent patterns/profiles is unknown, and multiple model fitting and testing is required to determine the optimal number of classes. However, under a Bayesian nonparametric framework, we are able to rely on dynamic and data-driven algorithms to identify an interpretable set of neighbourhood SDoH (NSDoH) profiles via a reproducible analytical workflow, while maintaining the flexibility to incorporate prior information into parameter estimation (Wade, 2022). These models have found great utility in nutrition, environmental health, and population genomics (Gao et al., 2024; Maduekwe et al., 2023; Medvedovic et al., 2004; B. J. Stephenson et al., 2020; B. J. K. Stephenson et al., 2024; Wang et al., 2017; Wu et al., 2024). Yet, none have been applied to area-level exposures of SDoH.

In this study, we aim to generate and characterize new NSDoH profiles to better understand their impact on EC care in Massachusetts (MA). Given binarized aggregate proportions of area-level SDoH variables from the ACS, we will implement a Bayesian multivariate Bernoulli mixture model (MBMM) to cluster neighbourhoods that share similar SDoH characteristics. Neighbourhoods are established based on census tract identifiers, as defined by the American Community Survey from 2015 to 2019. Once identified, these profiles will be assigned to patients from the Massachusetts Cancer Registry (MCR), based on their residential data, and analysed to determine if the care received was associated with their residential neighbourhood.

We organize the remaining sections of this article as follows. In Section 2, we describe our methodological approach, introducing the MBMM. In Section 3, we apply the MBMM to our census-tract level data and examine its association with EC treatment. In Section 4, we discuss the results and implications of this new approach to the field.

## 2 Methods

### 2.1 Data source

We obtained census tract (neighbourhood) level aggregate data from the 2015–2019 5-year ACS estimates for the state of MA. The data contained a total of 1,478 census tracts, which are hereafter referred to as neighbourhoods, that included approximately 6.8 million persons. Based on previous research, we selected 14 variables as indicators of NSDoH (Boscoe et al., 2021; Kolak et al., 2020). Given that the selected NSDoH variables were aggregate proportions and the Bernoulli distribution models binary data, we dichotomized each variable based on whether that feature fell above (*x*_*ij*_ = 1) or below (*x*_*ij*_ = 0) the state median value (Table 1). Dichotomization using the median was chosen due to the skewed distribution of several variables, which would have made other cutoffs (e.g. mean or quantile-based groupings) less interpretable or potentially biased by extreme values (online supplementary material, Supplementary Figure 1.1). The median provides a robust, nonparametric threshold that ensures a balanced comparison between neighbourhoods with relatively higher and lower exposures. Neighbourhoods above the state median threshold were classified as high exposure; those below were classified as low exposure. Data were extracted from respective ACS tables via the tidycensus package implemented in the R software environment (Walker & Herman, 2024).

The 14 variables selected for analysis fall within four thematic domains: (1) housing conditions and resources (Bowen & Mitchell, 2016; Mehdipanah et al., 2017; Munford et al., 2020); (2) economic security (Berg et al., 2021; Broer et al., 2019; Lindberg et al., 2022; Lut et al., 2021), (3) educational attainment, and (4) social and community context (Berg et al., 2021; Kolak et al., 2020). Housing conditions and resources are described by the proportion of neighbourhood households that were renter-occupied, lacked access to a vehicle, lacked complete plumbing, and experienced household crowding. Economic security is described by the proportion of households earning below the state median family income, participating in federal assistance [e.g. food stamps or Supplemental Nutrition Assistance Program (SNAP)], as well as the unemployment rate, working-class status, and female head of households. Educational attainment is described by the proportion of residents with no high school diploma. Social and community context is described by the dynamic interplay between multiethnic communities and the social structures that shape daily life (Sentell & Braun, 2012; Twersky et al., 2024). We include these indicators intentionally as they can be considered proxies for racism, xenophobia, and bias, which would impact a resident’s access to resources, opportunities, and social standing (Chant, 2004; Krieger et al., 1999; Lett et al., 2022). These indicators include the proportion of households with limited English (EN) proficiency and the proportion of residents identifying as Hispanic/Latino, non-Hispanic Black, and non-Hispanic Asian. To maintain interpretability across NSDoH variables, variables were recoded to reflect indicators of greater socioeconomic disadvantage. For example, the median family income variable was recoded such that a value of (*x*_*ij*_ = 1) represents low income (i.e. below the state median).

### 2.2 Multivariate Bernoulli mixture model

NSDoH profiles were identified using a fully Bayesian estimation of a MBMM (Papastamoulis & Rattray, 2017). This model clusters a population based on shared responses to a set of binary exposures. Let *i* ∈ {1, …, *n*} index an individual census tract, where *n* is the total number of neighbourhoods in MA. Let **x**_**i**_ = {*x*_*i*,1_, .., *x*_*i*,*p*_} denote a vector of observed binary indicators, where *x*_*i*,*j*_ = 1 denotes a high exposure to social determinant *j* ∈ {1, .., *p*} in census tract *i*. Let *K* denote the number of NSDoH profiles in the population. In practice, this number is typically not known *a priori*. To determine an appropriate number of profiles in the model, we overfit the model with *K*_max_ clusters, which over exceeds the true number of clusters. This approach, coupled with a prior that treats the number of clusters *K* < *K*_max_ as an unknown parameter, allows the observed data to drive the number of nonempty clusters estimated at the end of the Bayesian sampling algorithm (Papastamoulis, 2018; van Havre et al., 2015; Wade, 2022). The observed likelihood for the set of binary data **X** = {0, 1}^*n*×*p*^ under the MBMM is given by

(1) $$ℒ(\pi ,\theta |X)=\prod_{i=1}^{n}\left\{\sum_{k=1}^{K}\pi_{k}\prod_{j=1}^{p}\theta_{j|k}^{x_{i,j}}{\left(1-\theta_{j|k}\right)}^{1-x_{i,j}}\right\},$$

where ***π*** = {*π*_1_, …, *π*_*K*_} is the probability vector for membership of a given neighbourhood into one of the *K* NSDoH profiles. The probability matrix ***θ*** = {*θ*_1|1_, …, *θ*_*j*|*k*_} ^*p*×*K*^ summarizes the set of individual SDoH variables, where *θ*_*j*|*k*_ is the probability of a high exposure to variable *j* given the neighbourhood’s assignment to NSDoH profile *k*.

For estimation of these parameters, we augment the data by introducing a latent allocation variable ***z***_***i***_, such that *P*(*z*_*i*_ = *k*) = *π*_*k*_. Therefore, we consider the complete data {*x*_*i*_, *z*_*i*_} likelihood of the MBMM for computation:

(2) $${ℒ}^{c}(\pi ,\theta |X,Z)=\prod_{i=1}^{n}\prod_{k=1}^{K}{\left\{\pi_{k}\prod_{j=1}^{p}\theta_{j|k}^{x_{i,j}}{\left(1-\theta_{j|k}\right)}^{1-x_{i,j}}\right\}}^{I\left(z_{i}=k\right)}.$$

#### 2.2.1 Posterior computation

Model parameters are estimated via a Metropolis-coupled Markov chain Monte Carlo (MC^3^) algorithm described and implemented by Panagiotis Papastamoulis and Magnus Rattray as the R package BayesbinMix (Papastamoulis & Rattray, 2017). An allocation sampler is used to determine the most probable number of NSDoH profiles by placing a discrete prior on *K* up to a specified maximum *K*_max_. This approach allows the sampler to learn the optimal number of clusters directly from the data during posterior sampling, favouring parsimony while remaining unconstrained by a fixed number of components *a priori*. We identify the optimal number of clusters based on the maximum *a posteriori* number of nonempty clusters from the posterior distribution, *K*_map_. Once *K*_map_ is determined, we perform conditional inference to calculate the posterior mean of each model parameter. These posterior estimates are then used to describe each NSDoH profile *k*, based on the posterior distribution of ***θ***_·|***k***_ = (*θ*_1|*k*_, …, *θ*_*p*|*k*_), and assigning each MA census tract to the profile with the greatest posterior probability of membership.

We assume no prior knowledge of parameter values. To ensure flexibility in capturing the complex, heterogeneous patterns of census tract-level data, we set a relatively large upper bound on the number of clusters, *K*_max_ = 50, which intentionally exceeds the number of clusters we expect to identify in this data setting, which avoids underfitting.

The following priors were imposed on the model parameters to allow the data to drive estimation:

$$K|K_{\text{max}}\sim \text{Poisson}(\lambda =1)\text{truncated}\;\text{on}\;\text{the}\;\text{set}\left\{1,\ldots ,K_{\text{max}}\right\}$$

$$\pi |K\sim \text{Dirichlet}\left(\gamma_{1},\ldots ,\gamma_{K}\right),\;\text{where}\;\gamma_{k}=1\forall \;k.$$

$$\theta_{j|k}|K\sim \text{Beta}(\alpha ,\beta )\;\text{where}\;\alpha =1=\beta \;\forall \;j,k.$$

The generated MCMC samples were postprocessed using the equivalence classes representatives (ECR) algorithm to overcome label-switching identifiability issues inherent in Bayesian mixture models (Papastamoulis, 2014). Further details on model fit, including sensitivity analyses using alternate priors for the number of components, and other cluster postprocessing procedures are provided in online supplementary material, Supplementary Material Section 1.

#### 2.2.2 Regression analysis

Patient-level data were obtained from the MCR of the Massachusetts Department of Public Health. A total of 2,412 records were collected for women, aged 18 years or older, and diagnosed with endometrial cancer between 2015 and 2017. Cases were identified using the International Classification of Diseases for Oncology, third edition (ICD-O3) primary site (C54.1) and morphology codes for endometrial carcinoma (World Health Organization, 2013). The main outcome variable was completion of recommended treatment according to the National Comprehensive Cancer Network (NCCN) guidelines as defined in the year of treatment for each patient. These guidelines are based on tumour, grade, and stage, incorporating a combination of surgery, chemotherapy, and radiation as necessary. Adherence to NCCN treatment guidelines was treated as a dichotomous variable, where we defined optimal care as the patient receiving therapy following NCCN guidelines. Patients were assigned to a respective MBMM-derived NSDoH profile based on their residential data collected at the time of diagnosis. Associations were measured by adjusted odds ratio via a Bayesian logistic regression model, which also adjusted for patient-level clinical and sociodemographic characteristics at time of diagnosis (year of diagnosis, age, insurance status, and initial point of care facility type). Supplementary analysis was conducted to examine the association between NSDoH profiles and the type of initial care facility (academic medical centre vs. other facilities). More details about the guidelines and regression results are provided in online supplementary material, Supplementary Materials Section 2.

The Yost-type SES index was constructed and compared to our derived NSDoH profiles, as well as its relationship to our health outcome, receipt of optimal care. Details of construction and results are provided in online supplementary material, Supplementary Materials Section 3.

All codes for the MBMM, data analysis, and data wrangling, including ACS datasets, are available on GitHub at https://github.com/cbrodriguez01/ecbayesbinmix. All statistical analyses were conducted using the R software environment (version 4.3.1), and R packages (not exhaustive) tidycensus, ggplot2, tidyverse, table1, BayesBinMix, coda, readxl, stringr, brms, Shiny, leaflet, tigris, psych, and sf. The MBMM computations were run on the Harvard University Faculty of Arts and Sciences Research Computing (FASRC) Cannon cluster.

## 3 Results

### 3.1 NSDoH profiles results

Table 1 shows the NSDoH variables selected from the 2015 to 2019 ACS survey and clustered to characterize neighbourhoods in MA. Neighbourhoods were mostly composed of owner-occupied households and high proportions of residents with at least a high school degree. On average, across all neighbourhoods, about 13% of households received federal assistance (i.e. SNAP) in the past 12 months, and 52% of the population aged 16 or older had nonwhite collar occupations.

**Figure 1.**
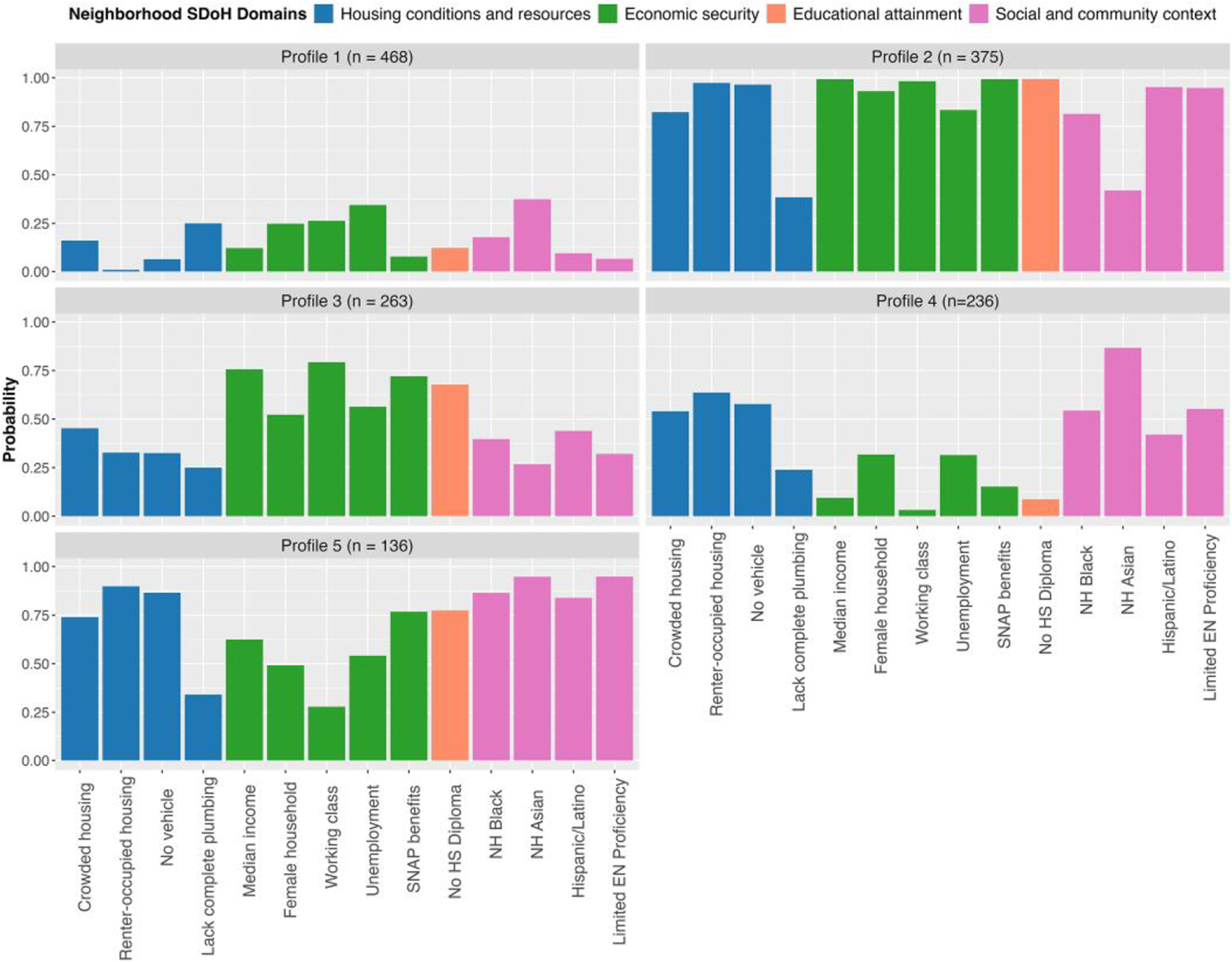
MBMM derived NSDoH profiles. Bars indicate the estimated posterior probability of high exposure to the NSDoH variable for a neighbourhood, given assignment to an NSDoH profile. SDoH variables are ordered/coloured by the NSDoH thematic domain. Abbreviations: HS = high school; EN = English; NH = Non-Hispanic; SNAP = Supplemental Nutrition Assistance Program. Shorthand names for NSDoH profiles: (1) advantaged NHW; (2) disadvantaged racially/ethnically diverse (BHL+), more renter-occupied housing with limited EN proficiency; (3) working class lower educational attainment; (4) racially/ethnically diverse (A+) and greater economic security and educational attainment; (5) racially/ethnically diverse (ABHL+), more renter-occupied housing with limited EN proficiency.

The model identified five NSDoH profiles, with a median profile assignment probability for neighbourhoods of 0.95 (IQR: 0.18). Figure 1 illustrates the NSDoH profile patterns derived from the MBMM. To facilitate interpretation, each profile was assigned a descriptive label reflecting the predominant NSDoH characteristics. Profile 1, *advantaged non-Hispanic White (NHW)*, represented about 32% of MA neighbourhoods with very low probabilities of exposure to poor NSDoH conditions across all domains of housing, employment, education, and social context (6.5%–37.4%). Specifically, this profile could be described as containing neighbourhoods mostly comprised of owner-occupied households, and median household incomes above or equal to the state’s median. Profile 2, *disadvantaged racially/ethnically diverse (BHL+; non-Hispanic Black (B) and Hispanic/Latino (HL)), more renter-occupied housing with limited EN proficiency*, represented the second largest cluster, containing 25% MA neighbourhoods. This profile exhibited characteristics of disadvantage and deprivation across all thematic domains. Neighbourhoods in Profile 2 had the highest probability of exposure to households with limited English proficiency (94.8%), female head of households (93.1%), household crowding (82.3%), renter-occupied housing (97.3%), no vehicle access (96.5%), working class occupations (98.2%), and higher proportions of all three ethnic minority groups, especially the proportion of non-Hispanic Black (81.4%) and Hispanic/Latino residents (95.2%). Profile 2 also contained neighbourhoods with more residents exposed to low economic security (federal assistance participation −99.3%; median household income below the state’s median −99.3%). Similar trends were found in the education domain, with all reporting no HS diploma (99.4%), compared to all other NSDoH profiles. Profile 3, *working class lower educational attainment*, favoured neighbourhoods with high probability of no high school diploma (67.7%), low probabilities of exposure to poor housing conditions and resources variables, and moderate proportions of ethnic minorities (26.8%–43.9%). Profile 4, *racially/ethnically diverse (A+;non-Hispanic Asian (A)) and greater economic security and educational attainment*, shared similar low exposures to disadvantages in the economic security and educational attainment domains with Profile 1, but differed across housing conditions and resources and social and community context. For example, Profile 4 had more neighbourhoods with a higher proportion of renter-occupied housing (63.6%) and residents identifying as non-Hispanic Asians (NHA;86.7%). Profile 5, *racially/ethnically diverse (ABHL+), more renter-occupied housing with limited EN proficiency*, favoured neighbourhoods with high probabilities of exposure to crowded and renter-occupied housing, less than HS education, and ethnic minorities (84%–95%). Compared to Profile 2, Profile 5 had a higher proportion of non-Hispanic Asians and less exposure to poor economic security.

The MBMM profile assignments for each census tract were recorded and linked with MA census tract geographic data. We developed a web-based interactive map using Shiny to display the MBMM results, available at https://mhn38j-carmen-rodriguez.shinyapps.io/nsdoh_profiles_app_urban/. The interactive application allows users to explore these spatial patterns in greater detail and is provided as a descriptive tool to complement the primary analytic results presented above. Visual inspection of the mapped profiles suggests that neighbourhoods sharing similar NSDoH profiles tend to cluster geographically and align with known patterns of urbanicity, immigration, and socioeconomic stratification across the state.

### 3.2 NSDoH profiles and receipt of optimal care for EC

Online supplementary material, Supplementary Table 2.1 in the Supplementary Materials Section 2 shows sociodemographic and clinical information of the analytical sample of EC patients with endometroid histology who received optimal care or NCCN guidelines adherent treatment (82.3%). These patients were predominantly non-Hispanic White (87.2%), foreign-born (49.7%), aged 50–64 (46.1%), privately insured (46.8%), diagnosed at stage I (91.5%), and with grade 1 tumours (52.9%). Patients were initially treated at large medical facilities (75.7%), academic medical centres (38.7%), and diagnosed by physicians specializing in family/internal medicine (47%).

Table 2 details the distribution of patient demographics based on the NSDoH profile at which they resided at the time of EC diagnosis. Race-ethnicity, birthplace, insurance status at diagnosis, and initial point of care facility characteristics were associated with NSDoH profiles. For example, among patients residing in neighbourhoods belonging to NSDoH Profile 2, 49% were foreign-born, 42.6% had Medicare or public insurance, and 37.6% were initially treated at academic medical centres.

**Figure 2.**
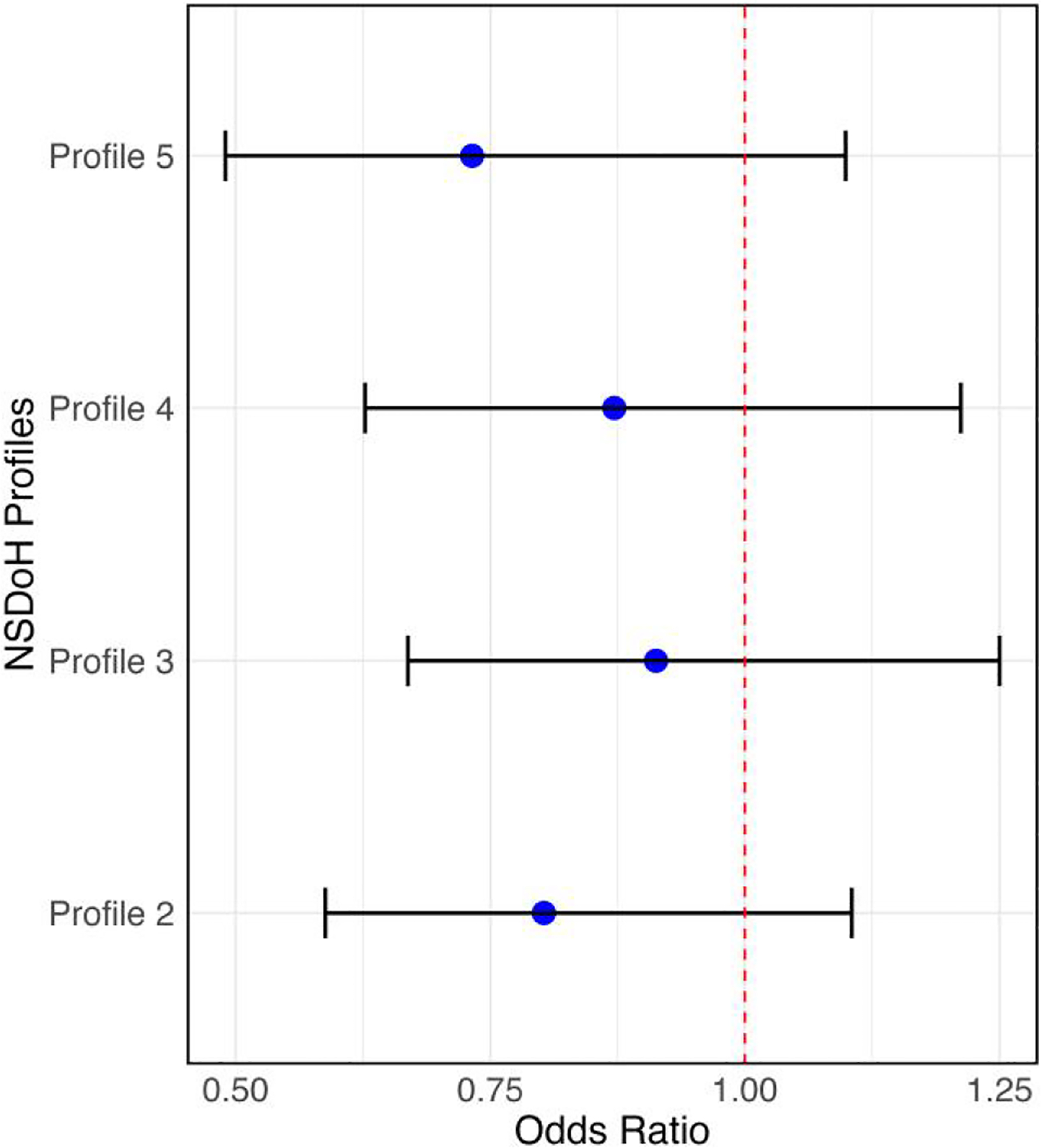
Adjusted odds of receiving optimal care by NSDoH profiles (Ref = NSDoH Profile 1; advantaged NHW) for patients diagnosed with EC from 2015 to 2019. Bars show 95% credible intervals. Shorthand names for NSDoH Profiles: (1) advantaged NHW, (2) disadvantaged racially/ethnically diverse (BHL+), more renter-occupied housing with limited EN proficiency, (3) working class lower educational attainment, (4) racially/ethnically diverse (A+) and greater economic security and educational attainment, and (5) racially/ethnically diverse (ABHL+), more renter-occupied housing with limited EN proficiency.

Figure 2 summarizes the results of the regression analysis. Although the 95% credible interval included the null value, patients in the NSDoH Profile 2 [disadvantaged racially/ethnically diverse (BHL+), more renter-occupied housing with limited EN proficiency] had lower odds [OR = 0.80, 95% credible interval (0.58,1.11)] of receiving optimal care compared to patients who resided in neighbourhoods belonging to the NSDoH Profile 1 (advantaged NHW), after adjusting for year of diagnosis, age at diagnosis, insurance status at diagnosis, and initial type of care facility.

We also examined the relationship between NSDoH profiles and the type of initial care facility (academic medical centre vs. other facilities). The results, adjusted for year, age, and insurance status at diagnosis, are detailed in online supplementary material, Supplementary Materials Section 2. Compared to NSDoH Profile 1 (advantaged NHW), patients who resided in NSDoH Profiles 4 [racially/ethnically diverse (A+) and greater economic security and educational attainment] and 5 [racially/ethnically diverse (ABHL+), more renter-occupied housing with limited EN proficiency] showed 95% credible intervals that excluded the null, indicating higher odds of receiving care at an academic medical centre, while those in NSDoH Profile 3 (working class lower educational attainment) had lower odds, though the credible interval included the null value.

## 4 Discussion

We used data from the ACS to derive NSDoH profiles using a fully Bayesian MBMM. To our knowledge, this is the first study to apply a Bayesian mixture model to characterize neighbourhood-level data in this context. This approach allowed us to estimate the appropriate number of profiles directly from the data, eliminating the need for *post hoc* testing. Our model identified five NSDoH profiles, which we characterized using neighbourhood SDoH variables belonging to four thematic domains: household conditions and resources, economic security, educational attainment, and social and community context.

We used these profiles to conduct a regression analysis to examine the association of NSDoH profiles and optimal care for EC. Our findings suggest some neighbourhood profiles experience lower odds of optimal EC care compared to patients in the advantaged NH White profile (Profile 1), though the 95% credible intervals included the null value. Specifically, patients residing in neighbourhood profiles with higher proportions of racially/ethnically diverse residents and greater housing and educational burden had lower odds of receiving optimal care. These findings are comparable to Rodriguez et al. (2021b) where they examined the association between neighbourhood socioeconomic status (NSES) using the Yost Index and adherence to the NCCN guidelines from 2006 to 2015. Consistent with our findings, patients residing in the most disadvantaged neighbourhoods with the lowest NSES had lower odds of receiving NCCN guideline-concordant care compared to those in the highest NSES group. In their study, 59.5% of patients received treatment adhering to NCCN guidelines, with the lowest adherence seen among Black, Latina, and American Indian/Alaska Native women (57.1%, 54.5%, and 52.7%, respectively). These proportions are lower than those in our study (82.3%) but comparable to a study using the Women’s Health Initiative (WHI), where they found 80% of patients received NCCN adherent treatment for EC (Felix et al., 2020). Our results focused specifically on MA, while their analysis covered the entire US. Additionally, similar to the Women’s Health Initiative, our patient population was predominantly non-Hispanic White and limited to individuals engaged in the healthcare system. This demographic composition may further obscure disparities in access to and receipt of guideline-concordant care. Systemic barriers such as insurance status, provider bias, and structural racism have contributed to lower levels of healthcare engagement among some racial and ethnic minority populations, resulting in delayed screening, diagnosis, and more advanced disease at presentation (Najor et al., 2023). This is also important, as differences in standard treatment regimens may exist for some groups due to variations in the histologic subtypes and stages of endometrial cancer they present with Stewart et al. (2023). Lastly, beyond the limitations of retrospective observational studies, the MCR lacks information on comorbidities, which may also impact differences in treatment adherence and recommendations. This could also explain the lack of guideline-concordant treatment rates for certain demographics.

Several studies have relied on the Yost index as a reliable measure of socioeconomic status at the neighbourhood level (Goel et al., 2023; Karia et al., 2023; Rodriguez et al., 2021a, 2021b). This measure of neighbourhood disadvantage uses a subset of our variables, which yields a different narrative of SES that is not necessarily reflective of the socioeconomic barriers of healthcare access. The Yost index is derived via a one component PCA or one factor analysis. This approach is a powerful and widely used method to reduce the dimensionality of high-dimensional datasets, but it suffers from interpretability issues because it outputs a linear combination of the original indicators (Jolliffe & Cadima, 2016). As demonstrated in our comparative analyses, this reliance on a composite score often obscures critical sociodemographic nuances that our profiles were better able to illustrate, specifically identifying multidimensional deprivation patterns across neighbourhoods, such as risks related to housing tenure and language access, that are frequently averaged out within a unidimensional index. Our approach, which relied on a mixture model framework, thus provides a holistic way to identify patterns of multiple interrelated exposures jointly. This approach provides interpretable clusters of neighbourhoods, making it easier to understand the heterogeneity of neighbourhood data, as well as the defining characteristics of each profile. The Bayesian framework of the MBMM provides the flexibility of estimating the model parameters using prior information, including the parameter describing the number of profiles (Miller & Harrison, 2018; Papastamoulis & Rattray, 2017; van Havre et al., 2015). Utilizing a Bayesian framework allowed us to borrow information within and across other neighbourhoods to improve the precision of our estimates, which improved the identifiability of our profiles.

This model is still met with limitations. First, model-based clustering is reflective of the data we use. In this study, we used 5-year estimates from the ACS from 2015 to 2019 for MA. Analysis using different ACS survey waves, different SDoH variables, or different geographies may yield different NSDoH profiles. For example, our results were based on binarized thresholds defined by median values for the state of MA. A different state would yield different cutoffs and, ultimately, different profiles. Second, the decision to dichotomize the SDoH variables was based on the skewed distributions found in the 2015–2019 cycles. This can sometimes result in a slight loss of information and reduced precision of the profile estimates since the full distribution of these variables was not considered. Future work should explore the development and implementation of a fully Bayesian multivariate beta mixture model that can flexibly accommodate bounded data, such as what we see in census-level data. Third, our study characterized associations between neighbourhood typologies and optimal receipt of healthcare among endometrial cancer patients who resided within these communities, thus the associations are not generalizable to the entire population. Studies like ours, which relies primarily on area-level data to define our neighbourhood profiles, provide a broader picture of the overall environment, resources, and barriers impacting the overall health and accessible care in that community (LaVeist et al., 2011). Future extensions of this research would benefit from access to multilevel datasets that link representative individual-level data with neighbourhood characteristics, allowing for the application of formal causal inference frameworks. Guided by frameworks such as the ‘place not race’ theory, future studies could use mediation analysis or qualitative methods to disentangle whether individual factors like insurance status act as mediators or confounders within specific contexts.

Complementary to these interpretive considerations are the technical limitations of the estimation process. Specifically, our two-stage approach treats the most probable neighbourhood assignment as an observed predictor, and this may induce attenuation bias. While our high posterior assignment probabilities suggest this bias is limited in our case, other studies may have less confident membership assignments. Joint modelling approaches, which account for latent class uncertainty, have been shown to mitigate such issues in fixed-class settings (Elliott et al., 2020). Future extensions of our Bayesian framework could integrate outcome information directly into the clustering process to formally propagate classification uncertainty through the inferential stage. Lastly, consistent with other similar studies, our analysis is cross-sectional (Lekkas et al., 2019) and does not account for the changes in neighbourhood demographics over time as a result of fair housing policies and gentrification. We focused our time frame on 2015–2019, which was based on the ACS 5-year estimates of data. Future studies should incorporate multiple survey cycles to assess how demographic shifts influence NSDoH profiles and, consequently, the access to and quality of healthcare and other outcomes for their residents.

Bayesian mixture models offer promising applications for neighbourhood-level data. Our approach enabled us to characterize and assign NSDoH profile patterns to neighbourhoods in MA. Geospatial mapping of NSDoH profiles demonstrated how we can leverage these tools to identify areas for targeted interventions. Using our NSDoH profiles to assess association with health outcomes, such as receipt of optimal care for EC, may give a more nuanced understanding of how SDoH overlap and co-occur within communities rather than in isolation to shape health experiences and outcomes. While this study focused on receipt of optimal care for EC patients, the derived profiles are translatable to a myriad of other outcomes and exploratory analyses.

## Supplementary Material

supplementary

Supplementary material is available online at Journal of the Royal Statistical Society: Series A.

## Figures and Tables

**Table 1.** Median estimate for MA for selected neighbourhood social determinants of health and ACS 2015–2019 table identification

NSDoH ACS variable (ACS table)	Median (IQR)
% of renter-occupied housing (DP04)	33.85 (41.83)
% households without a motor vehicle (DP04)	7.7 (14.52)
% crowding in household (DP04)^a^	1.28 (2.85)
% occupied housing units without complete plumbing (DP04)^b^	0 (0.5)
Estimate median household income in the past 12 months (inflation-adjusted; B19013)^c^	82,265 (47,359)
% female single-parent households with children younger than 18 (DP02)	3.9 (5.2)
% with food stamp/SNAP benefits in the past 12 months (DP03)	7.7 (12.9)
% unemployed/unemployment rate (DP03)	2.9 (2.2)
% employed population aged 16 years or older, working class (C24010)	53.9 (23.2)
% population aged 25 years or older with no high school diploma (DP02)	6.5 (10.2)
% language other than English: speak English less than ‘very well’ (DP02)	5.8 (11.4)
% Hispanics or Latinos (DP05)	5.95 (12.03)
% non-Hispanic Black (DP05)	2.8 (6.43)
% non-Hispanic Asian (DP05)	3.2 (7.8)

*Note*. IQR = interquartile range.

a Occupied housing units with 1.01 to 1.50 and 1.51 or more occupants per room/All occupied housing units for the same calendar year (CDC, 2023).

b We binarized this variable differently because the median was 0. Therefore, census tracts for which there were no households lacking complete plumbing were categorized as ‘0’, and the remaining tracts were categorized as ‘1’ to indicate lack of plumbing.

c Census tracts for which the median household income was ≥ to the state median were categorized as ‘0’, and remaining tracts were categorized as ‘1’ to indicate lower income.

**Table 2.** Sociodemographic and clinical characteristics of endometrial carcinoma cases between 2015 and 2017 in the Massachusetts Cancer Registry (MCR) by NSDoH profiles (*n* = 2,412)

	Neighbourhood SDoH profiles				
*n*(%)	Profile 1 *n* = 952	Profile 2 *n* = 439	Profile 3 *n* = 494	Profile 4 *n* = 340	Profile 5 *n* = 187
**Optimal care status**					
Not optimal	158 (16.6)	88 (20.0)	84 (17.0)	59 (17.4)	38 (20.3)
Optimal	794 (83.4)	351 (80.0)	410 (83.0)	281 (82.6)	149 (79.7)
**Race-ethnicity**					
Non-Hispanic White	910 (95.6)	286 (65.1)	459 (92.9)	313 (92.1)	135 (72.2)
Non-Hispanic Black	7 (0.7)	54 (12.3)	12 (2.4)	6 (1.8)	20 (10.7)
Hispanic	21 (2.2)	13 (3.0)	8 (1.6)	13 (3.8)	17 (9.1)
Other	11 (1.2)	82 (18.7)	9 (1.8)	7 (2.1)	10 (5.3)
**Birthplace**					
US-born	439 (46.1)	124 (28.2)	166 (33.6)	171 (50.3)	68 (36.4)
Foreign-born	458 (48.1)	217 (49.4)	298 (60.3)	144 (42.4)	84 (44.9)
Unknown	55 (5.8)	98 (22.3)	30 (6.1)	25 (7.4)	35 (18.7)
**Year of diagnosis (y)**					
2015	311 (32.7)	160 (36.4)	163 (33.0)	120 (35.3)	67 (35.8)
2016	345 (36.2)	156 (35.5)	172 (34.8)	121 (35.6)	61 (32.6)
2017	296 (31.1)	123 (28.0)	159 (32.2)	99 (29.1)	59 (31.6)
**Age at diagnosis (y)**					
Younger than 50	69 (7.2)	51 (11.6)	39 (7.9)	32 (9.4)	23 (12.3)
50–64	433 (45.5)	202 (46.0)	236 (47.8)	151 (44.4)	89 (47.6)
65 or older	450 (47.3)	186 (42.4)	219 (44.3)	157 (46.2)	75 (40.1)
**Insurance status at diagnosis**					
Private	463 (48.6)	132 (30.1)	204 (41.3)	177 (52.1)	88 (47.1)
Medicare	403 (42.3)	187 (42.6)	203 (41.1)	126 (37.1)	67 (35.8)
Public/Government	46 (4.8)	78 (17.8)	44 (8.9)	19 (5.6)	12 (6.4)
Other	27 (2.8)	38 (8.7)	38 (7.7)	12 (3.5)	16 (8.6)
Not insured	13 (1.4)	4 (0.9)	5 (1.0)	6 (1.8)	4 (2.1)
**Type of surgery received**					
None	28 (2.9)	19 (4.3)	20 (4.0)	13 (3.8)	11 (5.9)
Resection	920 (96.6)	415 (94.5)	471 (95.3)	327 (96.2)	176 (94.1)
Other surgery/Unknown	4 (0.4)	5 (1.1)	3 (0.6)	0 (0.0)	0 (0.0)
**Type of radiation administered**					
No radiation treatment	691 (72.6)	328 (74.7)	_367 (74.3)_	260 (76.5)	143 (76.5)
External beam radiation therapy (EBRT)	4 (0.4)	4 (0.9)	2 (0.4)	3 (0.9)	5 (2.7)
Brachytherapy	174 (18.3)	65 (14.8)	80 (16.2)	46 (13.5)	18 (9.6)
Other	83 (8.7)	42 (9.6)	45 (9.1)	31 (9.1)	21 (11.2)
**Chemotherapy status**					
No	833 (87.5)	381 (86.8)	443 (89.7)	292 (85.9)	152 (81.3)
Yes	119 (12.5)	58 (13.2)	51 (10.3)	48 (14.1)	35 (18.7)
**Stage at diagnosis**					
Stage I	806 (84.7)	361 (82.2)	416 (84.2)	278 (81.8)	142 (75.9)
Stage II	88 (9.2)	42 (9.6)	42 (8.5)	39 (11.5)	25 (13.4)
Stage III	32 (3.4)	11 (2.5)	13 (2.6)	11 (3.2)	9 (4.8)
Stage IV	26 (2.7)	25 (5.7)	23 (4.7)	12 (3.5)	11 (5.9)
**Grade at diagnosis**					
Grade 1	442 (46.4)	229 (52.2)	253 (51.2)	155 (45.6)	87 (46.5)
Grade 2	340 (35.7)	119 (27.1)	158 (32.0)	125 (36.8)	61 (32.6)
Grade 3	170 (17.9)	91 (20.7)	83 (16.8)	60 (17.6)	39 (20.9)
**Initial point of care facility type**					
Academic Medical Centers	347 (36.4)	165 (37.6)	163 (33.0)	168 (49.4)	100 (53.5)
Community	395 (41.5)	125 (28.5)	161 (32.6)	96 (28.2)	45 (24.1)
Specialty	16 (1.7)	6 (1.4)	3 (0.6)	3 (0.9)	3 (1.6)
Teaching	168 (17.6)	126 (28.7)	151 (30.6)	72 (21.2)	39 (20.9)
**Initial point of care facility size**					
Small (<100)	35 (3.7)	7 (1.6)	15 (3.0)	2 (0.6)	2 (1.1)
Medium (100–299)	257 (27.0)	67 (15.3)	97 (19.6)	73 (21.5)	32 (17.1)
Large (300+)	660 (69.3)	365 (83.1)	382 (77.3)	265 (77.9)	153 (81.8)
**Initial point of care facility, doctor specialty**					
Family/Internal medicine	451 (47.4)	198 (45.1)	170 (34.4)	193 (56.8)	106 (56.7)
Hematology	13 (1.4)	2 (0.5)	2 (0.4)	5 (1.5)	2 (1.1)
Gynecology and obstetrics	201 (21.1)	88 (20.0)	115 (23.3)	62 (18.2)	16 (8.6)
Oncology	136 (14.3)	69 (15.7)	84 (17.0)	35 (10.3)	24 (12.8)
Radiology	28 (2.9)	9 (2.1)	16 (3.2)	7 (2.1)	4 (2.1)
Other specialty	18 (1.9)	5 (1.1)	11 (2.2)	10 (2.9)	8 (4.3)
Unknown	105 (11.0)	68 (15.5)	96 (19.4)	28 (8.2)	27 (14.4)

## Data Availability

The R code used for this manuscript has been made publicly available in GitHub, https://github.com/cbrodriguez01/ecbayesbinmix. This also includes all the American Community Survey data we used for the multivariate Bernoulli mixture model, which is also publicly available at https://www.census.gov/programs-surveys/acs/data.html. The data that support the findings from the regression analysis in this study are available from the Massachusetts Cancer Registry-Massachusetts Department of Public Health (MCR-MDPH), but restrictions apply to the availability of MCR data, which were used under license for the current study, and so are not publicly available. Data are, however, available from the authors upon reasonable request and with permission of MCR-MDPH.
