# Supplementary material for: A Bayesian mixture model approach to examining neighbourhood social determinants of health in endometrial cancer care in Massachusetts
Source: J R Stat Soc Ser A Stat Soc. Author manuscript; Available in PMC 2026 May 20. (PMC13186426; doi:10.1093/jrsssa/qnag047)
Supplement: supplementary [file NIHMS2171872-supplement-supplementary.pdf]

Web-based supplemental materials for "A Bayesian Mixture Model Approach to Examining Neighborhood Social Determinants of Health Disparities in Endometrial Cancer Care in Massachusetts"

Carmen B Rodríguez, Stephanie M Wu, Stephanie Alimena, Alecia J McGregor and Briana JK Stephenson

1 Multivariate Bernoulli Mixture Model

Figure 1.1 shows the distribution of all the NSDoH variables. The distribution of these variables is skewed; therefore, as indicated by the vertical red line, we chose to dichotomize all variables for the MBMM model based on the median. We use the median because it is a robust non-parametric measure of central tendency unaffected by outliers. It ensures an equal data split, creating two comparison groups balanced in sample size.

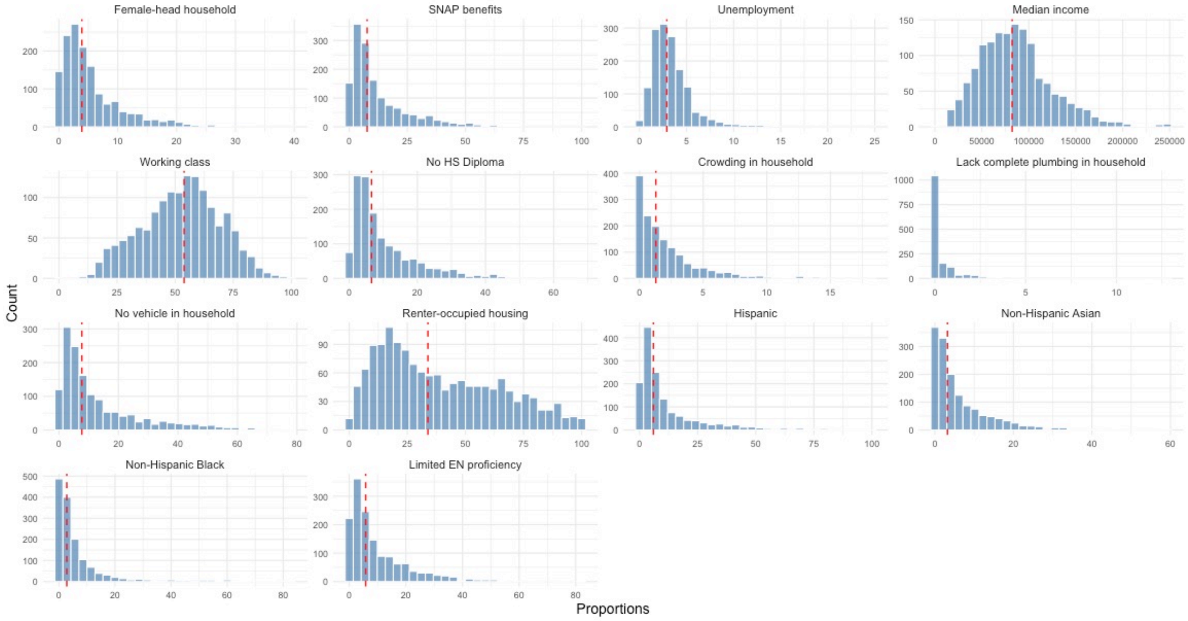

Supplementary Figure 1.1: Distributions of NSDoH variables from ACS 2015-2019 5-year estimates for Massachusetts.

Figure 1.2 shows the pairwise correlations of the selected NSDoH variables. Given that we used a small geographic unit (i.e., census tracts), some variables are highly correlated. In the case of the MBMM, the variance-covariance matrix of a mixture component of independent Bernoulli distributions is not diagonal and does not assume independence between variables within clusters; therefore, this model can accommodate neighborhood-level data.

We assumed the observed data comes from a mixture of K independent Bernoulli distributions. Here, K is the number of mixture components (i.e., clusters)[1] included in the model, while the true number of clusters in the data is $K_0 < K$ and is determined by model estimation. That is, we assume that for a binary data matrix $\mathbf{X} = \{\mathbf{x_1}, ...\mathbf{x_n}\}$, each $\mathbf{x_i} = \{x_{i,1}, ..., x_{i,p}\}$ is such that $p(x_i) = \sum_{k=1}^{K} \pi_k \prod_{j=1}^{p} \theta_{j|k}^{x_{i,j}} (1-\theta_{j|k})^{1-x_{i,j}}$ as described in the methods Section 2.2, where $\boldsymbol{\pi} = \{\pi_1, ..., \pi_K\}$ is the probability vector for the cluster assignments (i.e., the probability that a census tract belongs to cluster $k \in \{1, ..., K\}$) and $\sum_{k=1}^{K} \pi_k = 1$, and the probability matrix $\boldsymbol{\theta} = \{\theta_{j|k}\}^{p \times K}$ represents the probability of a high level of exposure to NSDoH

[1]In the manuscript text, we refer to clusters as profiles.

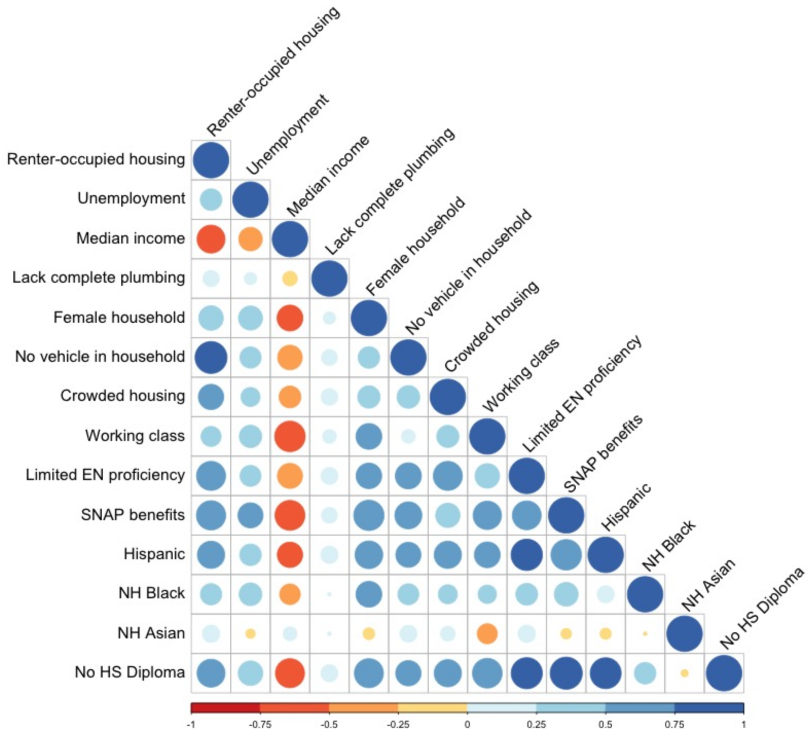

Supplementary Figure 1.2: Pairwise Pearson's Correlations of NSDoH variables from ACS 2015-2019.

variable j for a census tract given assignment to cluster k. Therefore, the observed likelihood for the specified model is

$$\mathcal{L}(\boldsymbol{\pi}, \boldsymbol{\theta}|\mathbf{X}) = \prod_{i=1}^{n} \sum_{k=1}^{K} \pi_k \prod_{j=1}^{p} \theta_{j|k}^{x_{i,j}} (1 - \theta_{j|k})^{1-x_{i,j}} \tag{1}$$

We augment the data by introducing a latent allocation variable $\boldsymbol{z_i}$, such that $z_i = k$ means that census tract i has been generated from the k-th cluster, and thus $P(z_i = k) = \pi_k$. For inference, we consider the complete data $\{x_i, z_i\}$ likelihood for the MBMM:

$$\mathcal{L}^c(\boldsymbol{\pi}, \boldsymbol{\theta}|\mathbf{X}, \mathbf{Z}) = \prod_{i=1}^{n} \prod_{k=1}^{K} \left\{ \pi_k \prod_{j=1}^{p} \theta_{j|k}^{x_{i,j}} (1 - \theta_{j|k})^{1-x_{i,j}} \right\}^{\mathbb{I}(z_i = k)} \tag{2}$$

The Bayesian framework for computation relies on three key data components: prior information about the model parameters, observed data likelihood, and posterior information. The prior information is defined as the first estimates of the distribution of the parameters before incorporating data through the Markov chain Monte Carlo (MCMC) sampler. As the sampler advances, both prior and observed data synergistically contribute to formulating insights about our target posterior distribution and updating information about the parameters using Bayes' Theorem.

Estimation of the parameters for the MBMM was performed using a Bayesian sampler described and implemented by Panagiotis Papastamoulis and Magnus Rattray as the R package `BayesbinMix` (Papastamoulis and Rattray, 2017). The main function in the package is `coupleMetropolis()`, which embeds an allocation sampler (Nobile and Fearnside, 2007) with an unknown number of mixture components (i.e., a

way to estimate the optimal number of components simultaneously) in a Metropolis-coupled Markov chain Monte Carlo (MC^3) algorithm. The MC^3 strategy is adopted to improve MCMC sampling by considering heated versions of the original target distribution. The function `coupleMetropolis()` takes as input the binarized observed data matrix $\mathbf{X}$, an upper bound on the number of clusters, defined in the function as K_{max}, prior information for all model parameters, and other inputs for computational efficiency. It then outputs the estimated posterior distribution of the model parameters $(\boldsymbol{\pi}, \boldsymbol{\theta})$ and the most probable number of NSDoH profiles defined as (K_{map}).For detailed information about this approach, please see their article *BayesBinMix: an R Package for Model-Based Clustering of Multivariate Binary Data* (Papastamoulis and Rattray, 2017).

We assume no prior knowledge of the initialization of the parameters. Consequently, all parameters are initialized with non-informative priors detailed below. The true number of NSDoH profiles is unknown. Using the upper bound K_{max}(also referred to as K in our model description above), we impose an overfitted finite mixture model(van Havre et al., 2015; Papastamoulis, 2018). We fit the model with a large upper bound on the number of clusters, $K = 50$, coupled with a prior on the number of clusters $K < K_{max}$, where the model treats K as another unknown parameter, and allow a data-driven approach to estimating K(van Havre et al., 2015; Wade, 2022; Papastamoulis, 2018). We impose the following priors on model parameters:

$$K|K_{max} \sim \text{Poisson}(\lambda = 1) \text{ truncated on the set}\{1, ..., K_{max}\}$$

For the other model parameters, we assume the following priors:

$$\boldsymbol{\pi} \mid K \sim \text{Dirichlet}(\gamma_1, \ldots, \gamma_K), \quad \text{where } \gamma_k = 1 \;\forall\; k.$$

$$\theta_{j|k}|K \sim Beta(\alpha, \beta) \quad \text{where } \alpha = 1 = \beta \;\forall\; j, k$$

The following full conditional distributions were used to update the model parameters:

$$\boldsymbol{\pi}|K, \mathbf{Z} \sim \text{Dirichlet}\left(\gamma_1 + \sum_{i=1}^{n} \mathbb{I}(z_i = 1), ..., \gamma_K + \sum_{i=1}^{n} \mathbb{I}(z_i = K)\right)$$

$$\theta_{j|k}|K, \mathbf{X}, \mathbf{Z} \sim \text{Beta}\left(\alpha + \sum_{i=1}^{n} \mathbb{I}(z_i = k)x_{i,j}, \beta + \sum_{i=1}^{n} \mathbb{I}(z_i = k) - \sum_{i=1}^{n} \mathbb{I}(z_i = k)x_{i,j}\right)$$

$$P(z_i = k|K, \mathbf{x}_i, \boldsymbol{\pi}, \boldsymbol{\theta}) \propto \pi_k \prod_{j=1}^{p} \theta_{j|k}^{x_{i,j}} (1 - \theta_{j|k})^{1-x_{i,j}}$$

Given that they are smaller geographic units, some census tracts have missing information on some of the NSDoH variables (ranging from 14 to 24 census tracts with missing data across all variables). This model can handle missing data by imputing these values using the parameter's posterior mean estimates. The MC^3 for posterior computation was run for 15,000 iterations, with thinning every 10 iterations (i.e., retaining every 10th sample) and the first 5000 iterations removed as part of posterior samples post-processing. For the MCMC chain heating parameter denoted as $h_m, m = 2, ..., M$, where M is the total number of parallel chains, we used incremental heating where the heat of the m'th chain is $h_m = 1/[1 + \Delta T \times (m - 1)]$, and we tuned the parameter ΔT such that swaps between chains were accepted 20%- 60% of time (Altekar et al., 2004). Through this process, we found that four (4) heated chains for the MC^3 algorithm produced good mixing, and for this type of data, smaller $\Delta T = 0.01$ works better. Posterior mean estimates were calculated from the remaining 1000 iterations collected from the sampler's output. The generated MCMC samples were postprocessed using the Equivalence Classes Representatives (ECR) algorithm to overcome label-switching identifiability issues inherent in Bayesian mixture models(Stephens, 2000). The most probable number of clusters (K_{map}) given the data was inferred, and the NSDoH profile assignment probabilities for each census tract were subsequently estimated after reordering with the ECR algorithm given K_{map}. Table 1.1 presents the median and interquartile range (IQR) of the assignment probabilities for census tracts to their most probable profile, while Figure 1.2 illustrates the distribution of assignment probabilities across all NSDoH profiles given K_{map}. Some cluster assignment probabilities were relatively low; however, they still represented the highest probabilities among all cluster options for each census tract given K_{map}. These lower probabilities

Supplementary Table 1.1: MBMM estimated assignment probability of census tracts to the most probable profile.

**NSDoH Profile**	**Median (IQR)**
Profile 1	0.946 (0.161)
Profile 2	0.972 (0.0581)
Profile 3	0.923 (0.200)
Profile 4	0.874 (0.233)
Profile 5	0.865 (0.259)

suggest the presence of unobserved heterogeneity, potentially driven by unmeasured NSDoH variables that we did not include in our model. Final clusters were qualitatively described based on thematic domains to define the NSDoH profiles, as discussed in Section 3.1 of the manuscript.

Additionally, we conducted sensitivity analyses using alternative priors (and combinations of priors) available in the software package. Specifically, we considered $K \sim Uniform(1, K_{max})$, and $\boldsymbol{\pi}|K \sim \text{Dirichlet}(\frac{1}{K_{max}}, ..., \frac{1}{K_{max}})$. Under these priors, we observed poorer MCMC mixing, as indicated by lower swap acceptance probabilities between heated chains. The estimated number of clusters consistently exceeded an interpretable size, and included occasional singleton clusters. The emergence of singleton clusters is consistent with using a sparse Dirichlet prior on the mixing weights, which approximates a Dirichlet process and promotes the formation of smaller clusters.

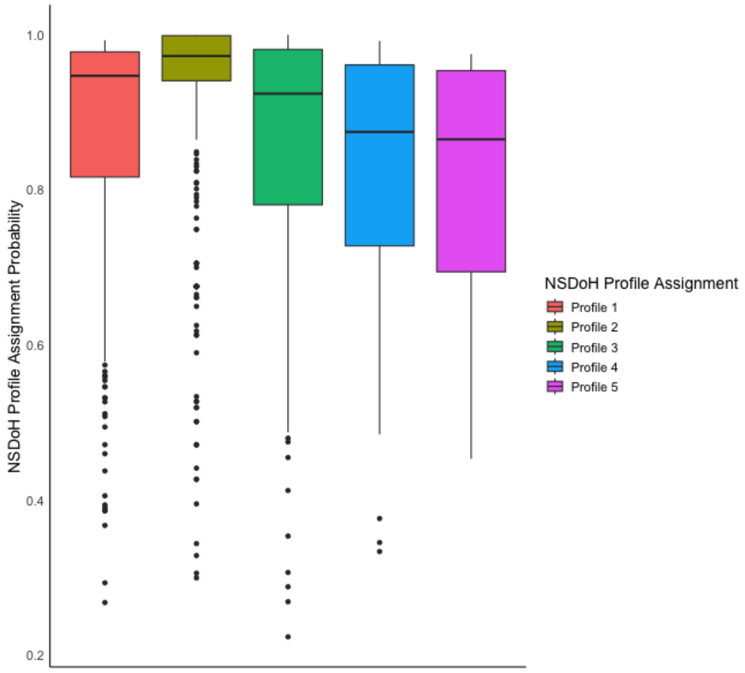

Supplementary Figure 1.3: Distribution of assignment probabilities for each NSDoH profile.

Table 1.2 shows the distribution of age and race/ethnicity within the census tracts used to further characterize NSDoH profiles in Section 3.2 of the manuscript.

Supplementary Table 1.2: Distribution of population characteristics within census tracts in a given NSDoH Profile.

	**Neighborhood SDoH Profiles**				
	**Profile 1**	**Profile 2**	**Profile 3**	**Profile 4**	**Profile 5**
**Mean (SD)**	**n = 468**	**n = 375**	**n= 263**	**n = 236**	**n = 136**
Census tract population	5060 (1860)	4220 (1710)	4520 (1800)	4500 (1810)	4770 (1660)
Median Age	45.6 (6.05)	35.3 (5.29)	43.3 (6.88)	38.0 (6.98)	34.6 (5.79)
Ages 20-24	5.35 (4.94)	8.10 (5.15)	7.19 (6.98)	7.78 (5.95)	10.8 (9.50)
Ages 25-34	9.12 (3.30)	16.3 (4.66)	12.3 (3.93)	20.4 (12.1)	24.0 (9.04)
Ages 35-44	11.4 (2.88)	13.1 (3.34)	11.4 (3.18)	12.8 (3.17)	12.8 (4.06)
Age 45-54	15.4 (3.15)	12.5 (3.14)	13.6 (3.42)	12.5 (4.22)	10.7 (3.93)
Age 55-59	8.29 (2.02)	6.06 (1.93)	7.91 (2.69)	6.09 (2.44)	5.48 (2.34)
Ages 60-64	7.66 (2.30)	5.44 (2.15)	7.38 (2.40)	5.47 (2.32)	5.10 (2.20)
Ages 65-74	11.4 (4.11)	7.15 (2.68)	10.7 (3.92)	8.55 (4.93)	7.21 (3.00)
Ages 75-84	5.51 (2.57)	3.59 (1.96)	5.42 (2.52)	4.35 (2.14)	3.60 (1.88)
Age 85 or older	2.55 (1.81)	1.74 (1.30)	2.72 (1.95)	2.28 (1.93)	2.02 (1.75)
Non-Hispanic White	89.0 (7.05)	41.9 (24.9)	82.0 (14.6)	74.7 (10.5)	57.0 (13.2)
Non-Hispanic Black	1.69 (2.22)	16.2 (19.0)	4.58 (6.93)	4.30 (4.93)	11.1 (9.91)
Non-Hispanic Asian	4.07 (4.99)	5.63 (9.05)	2.75 (4.12)	11.4 (8.04)	14.3 (9.65)
Hispanic or Latino	3.14 (2.58)	31.9 (22.4)	7.61 (9.18)	6.28 (3.86)	14.0 (9.48)

Shorthand names for NSDoH Profiles: 1) advantaged non-Hispanic White, 2) disadvantaged racially/ethnically diverse (BHL+; non-Hispanic Black (B) and Hispanic/Latino (HL)), more renter-occupied housing with limited EN proficiency, 3) working class lower educational attainment, 4) racially/ethnically diverse (A+; non-Hispanic Asian (A)) and greater economic security and educational attainment, 5) racially/ethnically diverse (ABHL+), more renter-occupied housing with limited EN proficiency.

2 Regression Analysis Additional

The main outcome is optimal care, defined as adherence to National Comprehensive Cancer Network (NCCN) guidelines. These guidelines recommend a combination of therapies depending on the stage and grade of the tumor (Figure 2.1). We used year-specific guidelines spanning the study period due to gradual changes in NCCN treatment guidelines. We determined optimal care by comparing the treatment received and the treatment recommended by NCCN. For example, we know that surgery is the first course of treatment, and thus if a woman received surgery alone or surgery and other additional therapies, as shown in Figure 2.1, based on the stage and grade of their tumor, then she is classified as having received optimal care. We combined all the corresponding data to create a binary outcome variable, where:

$$Y_i = \begin{cases} 1 & \text{Received optimal care: patient received treatment following NCCN guidelines} \\ 0 & \text{Did not receive optimal care} \end{cases}$$

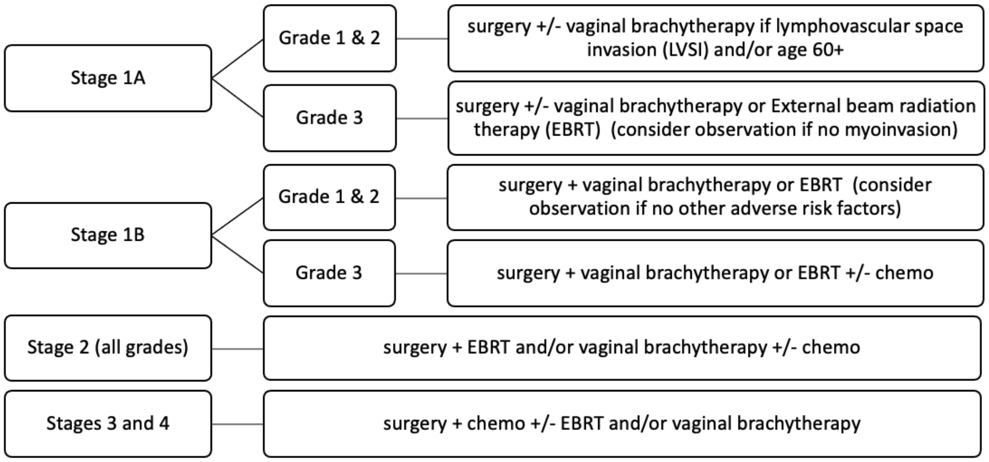

Supplementary Figure 2.1: National Comprehensive Cancer Network guidelines example from 2020.

Patient's sociodemographic characteristics included age at diagnosis (younger than 50 years old, 50-64 years, or 65 and older), health insurance status at diagnosis (private, Medicare, public/government, uninsured/other), nativity (U.S vs. foreign-born), and race/ethnicity (Hispanic, Non-Hispanic White, Non-Hispanic Black, Other race/ethnicity). Clinical characteristics included summary stage (distant, localized, regional), FIGO-stage (I,II,III,IV) using translations from the Summary Stage 2018 Coding Manual, tumor grade (1,2,3), and year of diagnosis. Treatment variables included type of surgery(none, resection, tumor destruction, other/unknown), type of radiation (brachytherapy, external beam (EBRT), other), and chemotherapy status (no, yes), and dates for each corresponding procedure. Additionally, we included information on the type (academic medical centers, community, specialty, and teaching) and size of the facility at the initial point of cancer management.

Table 2.1 shows the distribution of patient characteristics by optimal care status defined as NCCN treatment adherence. For all the Bayesian logistic regression analyses, we used the `brms` package with default non-informative priors designed to have minimal influence on the results. The model was run with 2,000 iterations per chain, including a 500-iteration burn-in. Two chains were used, and they showed good mixing for all parameters, indicating no evidence of non-convergence. Table 2.2 shows the regression results shown in the main text in table format. Effect sizes remained stable between Model 1 and Model 2, indicating that the association between neighborhood profiles and optimal care is not primarily driven by individual-level demographics or facility characteristics.

Supplementary Table 2.1: Sociodemographic and Clinical Characteristics of Massachusetts Cancer Registry Endometrial Cancer Cases from 2015-2017 by Optimal Care Status (n=2412).

	**Received optimal care (n=1985)**	**Did not receive optimal care (n= 427)**
**Race-Ethnicity**		
Non-Hispanic White	1739 (87.6)	364 (85.2)
Non-Hispanic Black	75 (3.8)	24 (5.6)
Hispanic	58 (2.9)	14 (3.3)
Other	99 (5.0)	20 (4.7)
**Birthplace**		
US-born	796 (40.1)	172 (40.3)
Foreign-born	986 (49.7)	215 (50.4)
Unknown	203 (10.2)	40 (9.4)
**Year of diagnosis (y)**		
2015	667 (33.6)	154 (36.1)
2016	723 (36.4)	132 (30.9)
2017	595 (30.0)	141 (33.0)
**Age at diagnosis (y)**		
Younger than 50	181 (9.1)	33 (7.7)
50-64	954 (48.1)	157 (36.8)
65 or older	850 (42.8)	237 (55.5)
**Insurance Status at Diagnosis**		
Private	928 (46.8)	136 (31.9)
Medicare	769 (38.7)	217 (50.8)
Public/Government	152 (7.7)	47 (11.0)
Other	110 (5.5)	21 (4.9)
Not insured	26 (1.3)	6 (1.4)
**Type of surgery received**		
None	0 (0.0)	91 (21.3)
Resection	1983 (99.9)	326 (76.3)
Other/Unknown	2 (0.1)	10 (2.3)
**Type of radiation administered**		
No radiation treatment	1558 (78.5)	231 (54.1)
External beam (EBRT)	14 (0.7)	4 (0.9)
Brachytherapy	367 (18.5)	16 (3.7)
Other	46 (2.3)	176 (41.2)
**Chemotherapy status**		
No	1828 (92.1)	273 (63.9)
Yes	157 (7.9)	154 (36.1)
**Stage at diagnosis**		
Stage I	1816 (91.5)	187 (43.8)
Stage II	54 (2.7)	182 (42.6)
Stage III	62 (3.1)	14 (3.3)
Stage IV	53 (2.7)	44 (10.3)
**Grade at diagnosis**		
Grade 1	1050 (52.9)	116 (27.2)
Grade 2	670 (33.8)	133 (31.1)
Grade 3	265 (13.4)	178 (41.7)
**Initial point of care facility type**		
Academic Medical Centers	768 (38.7)	175 (41.0)
Community	693 (34.9)	129 (30.2)
Specialty	15 (0.8)	16 (3.7)
Teaching	469 (23.6)	87 (20.4)
**Initial point of care facility size**		
Small (< 100)	39 (2.0)	22 (5.2)
Medium (100-299)	450 (22.7)	76 (17.8)
Large (300+)	1496 (75.4)	329 (77.0)
**Initial point of care facility , doctor specialty**		
Family/Internal medicine	933 (47.0)	185 (43.3)
Hematology	12 (0.6)	12 (2.8)
Gynecology & obstetrics	407 (20.5)	75 (17.6)
Oncology	305 (15.4)	43 (10.1)
Radiology	45 (2.3)	19 (4.4)
Other specialty	44 (2.2)	8 (1.9)
Unknown	239 (12.0)	85 (19.9)

Supplementary Table 2.2: Association between NSDoH Neighborhood Profiles and Receipt of Optimal Care for EC.

	Model 1		Model 2	
	**OR**	**95% Credible Interval**	**OR**	**95% Credible Interval**
**Neighborhood SDoH Profile**				
Profile 1	Referent		Referent	
Profile 2	0.79	(0.59, 1.07)	0.80	(0.59, 1.11)
Profile 3	0.97	(0.73, 1.31)	0.91	(0.67, 1.25)
Profile 4	0.95	(0.68, 1.31)	0.87	(0.63, 1.21)
Profile 5	0.79	(0.53, 1.17)	0.73	(0.49, 1.09)

Model 1: Unadjusted model.

Model 2: Adjusted for age, year, insurance status at diagnosis, and type of initial care facility.

Shorthand names for NSDoH Profiles: 1) advantaged non-Hispanic White, 2) disadvantaged racially/ethnically diverse (BHL+), more renter-occupied housing with limited EN proficiency, 3) working class lower educational attainment, 4) racially/ethnically diverse (A+) and greater economic security and educational attainment, 5) racially/ethnically diverse (ABHL+), more renter-occupied housing with limited EN proficiency.

We also explored the association between NSDoH profiles and the type of facility where patients were diagnosed or received treatment. Due to data limitations, we could not distinguish between these scenarios. For this sub-analysis, we dichotomized the facility type as an academic medical center versus other types. Similarly to the optimal care analysis, we conducted a Bayesian logistic regression adjusted for age, year, and insurance status at diagnosis, with results in Table 2.3. Even after adjustment, NSDoH profiles 4 (racially/ethnically diverse (A+) and greater economic security and educational attainment) and 5 (racially/ethnically diverse (ABHL+), more renter-occupied housing with limited EN proficiency) were linked to higher odds of receiving care at academic centers. The interactive map in Section 3.1 shows that neighborhoods in profiles 4 and 5 are often located near universities, which are in proximity to academic medical centers. In contrast, patients residing in neighborhoods in NSDoH profile 3 (working class lower educational attainment) had lower odds of receiving care at an academic facility, although the 95% Credible Interval included the null value. These patients were more likely to receive care at either a community or teaching hospital (Table 2), and from observing the interactive map for NSDoH profile 3, most neighborhoods may be located farther from academic medical centers, making it less convenient for residents to access these facilities. Despite lower probabilities of "no vehicle in the household" in this profile, transportation challenges may still be a barrier, particularly for working-class populations (for which there is a high probability of 79%) with limited flexibility in work schedules.

Supplementary Table 2.3: Univariate and Multivariate Logistic Regression of Receiving Care at an Academic Medical Center Among Endometrial Cancer Cases Between 2015 and 2017 in the Massachusetts Cancer Registry (n=2412).

	Model 1		Model 2	
	**OR**	**95% Credible Interval**	**OR**	**95% Credible Interval**
**Neighborhood SDoH Profile**				
Profile 1	Referent		Referent	
Profile 2	1.049	(0.835, 1.324)	1.116	(0.875, 1.424)
Profile 3	0.860	(0.674, 1.083)	0.883	(0.702, 1.101)
Profile 4	1.701	(1.328, 2.181)	1.697	(1.313, 2.203)
Profile 5	2.011	(1.482, 2.730)	2.033	(1.464, 2.825)

Model 1: Unadjusted model.

Model 2: Adjusted for age, year, and insurance status at diagnosis.

Shorthand names for NSDoH Profiles: 1) advantaged non-Hispanic White, 2) disadvantaged racially/ethnically diverse (BHL+), more renter-occupied housing with limited EN proficiency, 3) working class lower educational attainment, 4) racially/ethnically diverse (A+) and greater economic security and educational attainment, 5) racially/ethnically diverse (ABHL+), more renter-occupied housing with limited EN proficiency.

3 Comparative analysis to Yost Index (unidimensional socioeconomic index)

3.1 Construction of Yost Index

To compare the proposed multidimensional NSDoH profiles with a commonly used one-dimensional neighborhood socioeconomic index, we constructed a Yost-type socioeconomic status (SES) index following the definitions provided by SEER National Cancer Institute Surveillance, Epidemiology and End Results Program and used by authors (LaFantasie and Boscoe, 2022; Yost et al., 2001; Boscoe et al., 2021). We obtained data from 2015-2019 American Community Survey 5-year estimates at the census tract level using `tidycensus` R package, and derived seven variables reflecting income, employment, housing value, housing costs, poverty, educational attainment, and working-class composition. Definitions for these variables can be found at (National Cancer Institute Surveillance, Epidemiology and End Results Program).

The values of each of the seven variables in the Yost Index were converted to ranks in ascending order so that they were ordered from most affluent census tract to the least affluent. Maximum likelihood factor analysis was performed using the `psych` R package. The first principal component was extracted, which explained 46% of the total variance across all seven variables. This factor was dominated by income and poverty rate, which exhibited the highest standardized loadings. The resulting factor scores were converted into percentiles such that a value of 1 represented the most affluent percentile and 100 the least affluent. These percentiles were further categorized into quintiles consistent with SEER definitions(Yu et al., 2014).

3.2 Comparative results

Table 3.1 shows a cross-tabulation of the Yost-type SES quintiles and NSDoH profiles. While the Yost SES quintiles provide a measure of economic standing, the NSDoH profiles reveal significant sociodemographic heterogeneity within those strata. For instance, the "Lowest" SES quintile is predominantly captured by Profile 2, characterized by racial diversity (BHL+) and limited English proficiency. Conversely, the "Highest" SES quintile is split across the "Advantaged non-Hispanic White" profile (Profile 1) and "Racially diverse" profiles with high economic security (Profiles 4 and 5). This distribution underscores that neighborhoods with identical economic SES scores may face vastly different social and structural barriers, particularly regarding language and housing tenure.

Table 3.2 provides summaries of both adjusted and unadjusted odds ratios from Bayesian logistic regression models measuring the association of the Yost SES index and optimal care. In both models, Yost SES quintiles showed 95% posterior credible intervals including the null value, with only the lowest SES quintile exhibiting a modestly lower odds of optimal care relative to the highest quintile. Similar results were observed when outcome was treated as a continuous percentile score. These regression results are consistent with the results of the NSDoH profiles, however, while the SES index identifies a general economic gradient, the NSDoH profiles reveal that structural barriers, specifically housing instability and limited English proficiency, drive care disparities even in neighborhoods with moderate economic resources. Consequently, the multidimensional cluster approach seems to "uncover" risks in diverse, high-renter areas (Profiles 2 and 5) that a unidimensional SES index obscures by focusing primarily on income and poverty (Table 2.2). For reference, Table 2.2 shows the adjusted and unadjusted odd ratios and 95% credible intervals for the association between optimal care and NSDoH profiles.

Supplementary Table 3.1: Cross-tabulation of Yost-type socioeconomic status (SES) quintiles and NSDoH neighborhood profiles. Values are number (percentage) of census tracts.

	Overall	Profile 1	Profile 2	Profile 3	Profile 4	Profile 5
n	2412	952	439	494	340	187
**Yost SES Quintile, n (%)**						
Highest	544 (22.6)	274 (28.8)	1 (0.2)	99 (20.0)	104 (30.6)	66 (35.3)
High-middle	522 (21.6)	358 (37.6)	1 (0.2)	6 (1.2)	146 (42.9)	11 (5.9)
Middle	551 (22.8)	287 (30.1)	11 (2.5)	123 (24.9)	87 (25.6)	43 (23.0)
Lower-middle	466 (19.3)	33 (3.5)	137 (31.2)	237 (48.0)	3 (0.9)	56 (29.9)
Lowest	329 (13.6)	0 (0.0)	289 (65.8)	29 (5.9)	0 (0.0)	11 (5.9)

Shorthand names for NSDoH Profiles: 1) advantaged non-Hispanic White, 2) disadvantaged racially/ethnically diverse (BHL+), more renter-occupied housing with limited EN proficiency, 3) working class lower educational attainment, 4) racially/ethnically diverse (A+) and greater economic security and educational attainment, 5) racially/ethnically diverse (ABHL+), more renter-occupied housing with limited EN proficiency.

Supplementary Table 3.2: Association between Yost SES Quintiles and Receipt of Optimal Care for EC.

	Model 1		Model 2	
	**OR**	**95% Credible Interval**	**OR**	**95% Credible Interval**
**Yost SES Quintile**				
Highest	Referent		Referent	
High-middle	0.97	(0.71, 1.35)	0.95	(0.68, 1.31)
Middle	1.07	(0.77, 1.46)	1.07	(0.77, 1.46)
Lower-middle	0.99	(0.72, 1.38)	0.98	(0.70, 1.41)
Lowest	0.71	(0.50, 1.01)	0.73	(0.50, 1.04)

Model 1: Unadjusted model.

Model 2: Adjusted for age, year, insurance status at diagnosis, and type of initial care facility.

References

G. Altekar, S. Dwarkadas, J. P. Huelsenbeck, and F. Ronquist. Parallel Metropolis coupled Markov chain Monte Carlo for Bayesian phylogenetic inference. *Bioinformatics*, 20(3):407–415, Feb. 2004. ISSN 1367-4803. doi: 10.1093/bioinformatics/btg427.

F. P. Boscoe, B. Liu, and F. Lee. A comparison of two neighborhood-level socioeconomic indexes in the United States. *Spatial and Spatio-temporal Epidemiology*, 37:100412, June 2021. ISSN 1877-5845. doi: 10.1016/j.sste.2021.100412.

J. LaFantasie and F. Boscoe. Empirical Approach to Developing an Optimal Socioeconomic Status Index for Health Surveillance, Jan. 2022. Pages: 2022.01.14.22269310.

National Cancer Institute Surveillance, Epidemiology and End Results Program. Time-dependent county attributes. `https://seer.cancer.gov/seerstat/variables/countyattribs/time-dependent.html`.

A. Nobile and A. T. Fearnside. Bayesian finite mixtures with an unknown number of components: The allocation sampler. *Statistics and Computing*, 17(2):147–162, June 2007. ISSN 1573-1375. doi: 10.1007/s11222-006-9014-7.

P. Papastamoulis. Overfitting Bayesian mixtures of factor analyzers with an unknown number of components. *Computational Statistics and Data Analysis*, 124:220–234, Aug. 2018. ISSN 0167-9473. doi: 10.1016/j.csda.2018.03.007.

P. Papastamoulis and M. Rattray. BayesBinMix: an R Package for Model Based Clustering of Multivariate Binary Data. *The R Journal*, 9(1):403–420, 2017. ISSN 2073-4859.

M. Stephens. Dealing With Label Switching in Mixture Models. *Journal of the Royal Statistical Society Series B: Statistical Methodology*, 62(4):795–809, Nov. 2000. ISSN 1369-7412. doi: 10.1111/1467-9868.00265.

Z. van Havre, N. White, J. Rousseau, and K. Mengersen. Overfitting Bayesian Mixture Models with an Unknown Number of Components. *PLoS ONE*, 10(7):e0131739, July 2015. ISSN 1932-6203. doi: 10.1371/journal.pone.0131739.

S. Wade. Bayesian cluster analysis. *Philosophical transactions. Series A, Mathematical, physical, and engineering sciences*, 381(2247):20220149, 2022. ISSN 1364-503X. doi: 10.1098/rsta.2022.0149.

K. Yost, C. Perkins, R. Cohen, C. Morris, and W. Wright. Socioeconomic status and breast cancer incidence in California for different race/ethnic groups. *Cancer causes and control: CCC*, 12(8):703–711, Oct. 2001. ISSN 0957-5243. doi: 10.1023/a:1011240019516.

M. Yu, Z. Tatalovich, J. T. Gibson, and K. A. Cronin. Using a composite index of socioeconomic status to investigate health disparities while protecting the confidentiality of cancer registry data. *Cancer Causes & Control*, 25(1):81–92, 2014. doi: 10.1007/s10552-013-0310-2.
